# A systematic review of models of care for polycystic ovary syndrome highlights the gap in the literature, especially in developing countries

**DOI:** 10.3389/fendo.2023.1217468

**Published:** 2023-07-19

**Authors:** Eka Melson, Meri Davitadze, Kashish Malhotra, PCOS SEva working group, Aya Mousa, Helena Teede, Jacky Boivin, Mala Thondan, Chau Thien Tay, Punith Kempegowda

**Affiliations:** ^1^ Leicester Diabetes Centre, University of Leicester, Leicester, United Kingdom; ^2^ Department of Diabetes and Endocrinology, Clinic NeoLab, Tbilisi, Georgia; ^3^ Institute of Applied Health Research, University of Birmingham, Birmingham, United Kingdom; ^4^ Dayanand Medical College and Hospital, Punjab, India; ^5^ Monash Centre for Health Research and Implementation, Monash University, Melbourne, VIC, Australia; ^6^ School of Psychology, Cardiff University, Cardiff, Wales, United Kingdom; ^7^ Primary Care, Harp Family Medical Centre, Melbourne, VIC, Australia; ^8^ Queen Elizabeth Hospital, University Hospitals Birmingham NHS Foundation Trust, Birmingham, United Kingdom

**Keywords:** polycystic ovary syndrome, PCOS, model of care, multidisciplinary care, quality of life

## Abstract

**Introduction:**

The aim of the study was to identify available polycystic ovary syndrome (PCOS) models of care (MoCs) and describe their characteristics and alignment with the international PCOS guideline.

**Methods:**

Ovid MEDLINE, All EBM, PsycINFO, Embase, and CINAHL were searched from inception until 11 July 2022. Any study with a description of a PCOS MoC was included. Non-evidence-based guidelines, abstracts, study protocols, and clinical trial registrations were excluded. We also excluded MoCs delivered in research settings to minimize care bias. Meta-analysis was not performed due to heterogeneity across MoCs. We describe and evaluate each MoC based on the recommendations made by the international evidence-based guideline for assessing and managing PCOS.

**Results:**

Of 3,671 articles, six articles describing five MoCs were included in our systematic review. All MoCs described a multidisciplinary approach, including an endocrinologist, dietitian, gynecologist, psychologist, dermatologist, etc. Three MoCs described all aspects of PCOS care aligned with the international guideline recommendations. These include providing education on long-term risks, lifestyle interventions, screening and management of emotional well-being, cardiometabolic diseases, and the dermatological and reproductive elements of PCOS. Three MoCs evaluated patients’ and healthcare professionals’ satisfaction, with generally positive findings. Only one MoC explored the impact of their service on patients’ health outcomes and showed improvement in BMI.

**Conclusion:**

There is limited literature describing PCOS MoCs in routine practice. Future research should explore developing cost-effective co-created multidisciplinary PCOS MoCs globally. This may be facilitated by the exchange of best practices between institutions with an established MoC and those who are interested in setting one up.

**Systematic review registration:**

https://www.crd.york.ac.uk/prospero/display_record.php?RecordID=346539, identifier CRD42022346539.

## Introduction

**Figure 1:**
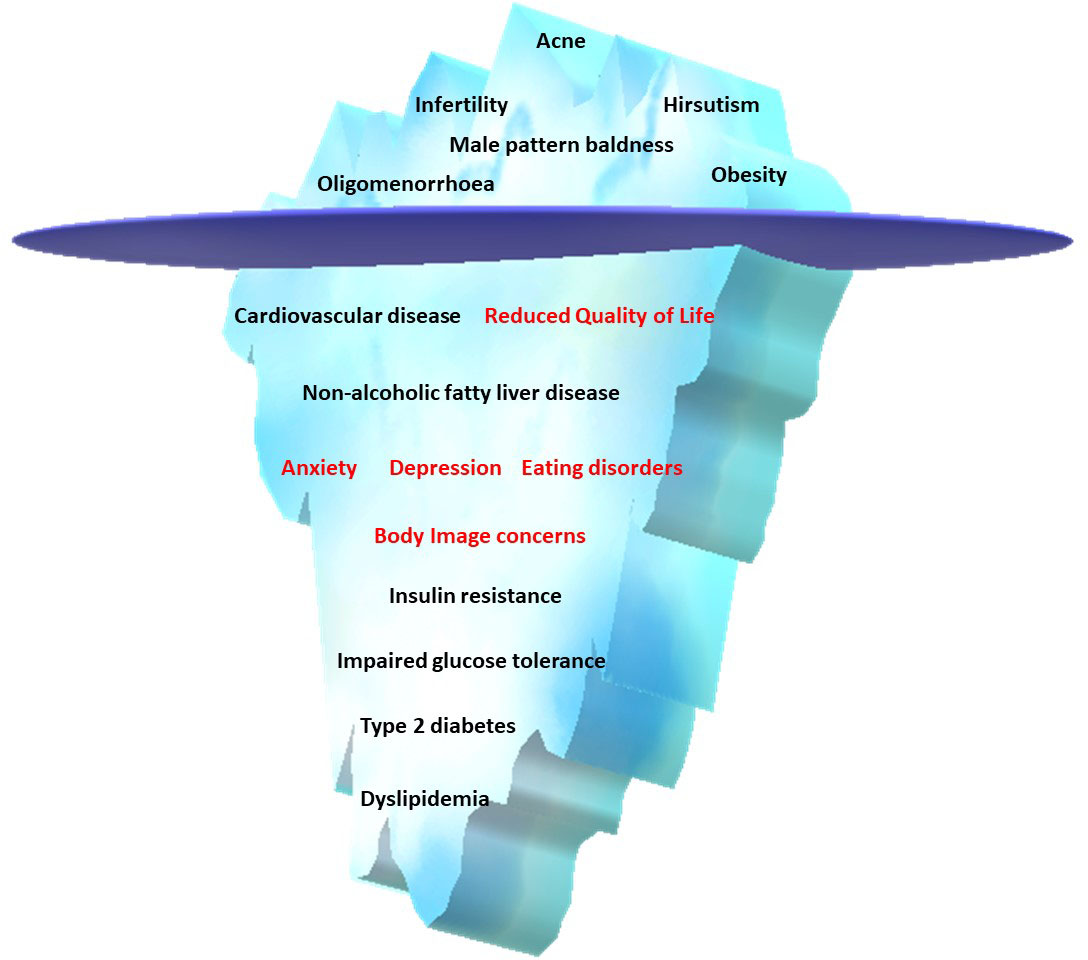
An iceberg phenomenon in polycystic ovary syndrome.

Polycystic ovary syndrome (PCOS) is one of the most common endocrinopathies among women of reproductive age, with a prevalence of 8%–13%, depending on the phenotype and the diagnostic criteria used (1). The diagnostic features of the disease are clinical and/or biochemical hyperandrogenism, oligo/anovulation, and a polycystic morphologic appearance of the ovaries (2, 3). PCOS was originally perceived as a reproductive disorder. However, mounting evidence suggests that PCOS is also a metabolic condition associated with overweight/obesity (4, 5), type 2 diabetes mellitus (T2DM) (6, 7), fatty liver disease (8–10), and cardiovascular disease (CVD) (11, 12). It also has a significant psychological burden that is more than just a consequence of the physical symptoms of PCOS (13–16). Kempegowda et al. proposed an ‘iceberg phenomenon’ to highlight the neglected and overlooked impact on various aspects of women’s and individuals; with PCOS, alongside potential reproductive dysfunction (**Figure 1**) (17).

Several studies have shown that women and individuals with PCOS often have a significant delay in diagnosis and are dissatisfied with their diagnostic experience, information provision, and the management of their PCOS (18–21). Qualitative research has shown that women and individuals with PCOS often felt they were not taken seriously by their doctors (22) and that care fell short of their expectations due to limited evidence-based treatment options (23). The International PCOS Guideline (24) recommends patient-centric models of care (MoC) that meet the needs of women and individuals with PCOS across the complexity of clinical features.

**Figure 2:**
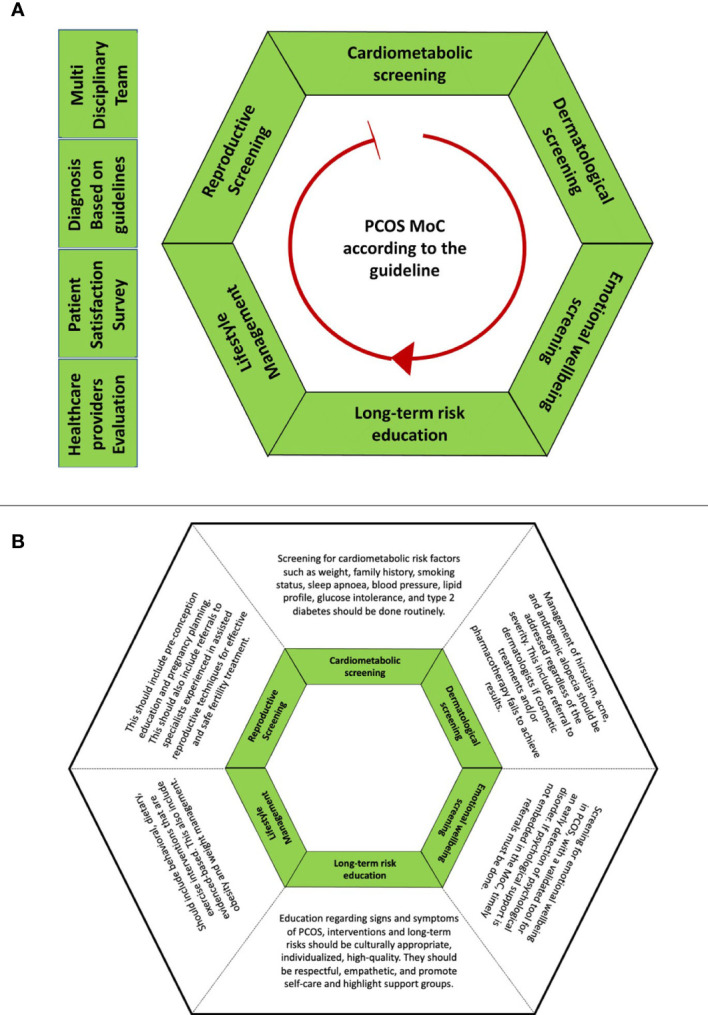
Detailed description of the best-practice PCOS MoC aligned with the international evidence-based guideline for the assessment and management of polycystic ovary syndrome (2018) (24). **(A)** outlines 10 recommendations arranged in alphabetical order. The red arrow is the pointer for the starting point of reading the hexagon at cardiometabolic screening. **(B)** outlines the descriptions for the components of MoC

An MoC is generally conceptualized as an overarching provision of care that is codesigned with end-users, may be shaped by a theoretical basis, and aligns with evidence-based practice and defined standards (25, 26). A holistic, best-practice PCOS MoC would entail access to primary care, endocrinologists, gynecologists, dermatologists, dieticians, and psychologists as required to educate women and individuals with PCOS about their condition and its long-term consequences, address cardiometabolic, reproductive, and dermatological issues, and provide lifestyle interventions and psychological and emotional support (**Figure 2**) (24). In the US and Australia, some MoCs have been implemented in accordance with the international guidelines. The involvement of a psychologist and cognitive-behavioral therapy in PCOS resulted in greater weight loss, improved quality of life, and reduced depression and anxiety (27). However, there is no literature comparing findings across MoCs to advance best practices that can be shared and adopted in other places where women and individuals with PCOS are managed. Furthermore, it is also important to consider the settings for the MoCs, as they can be significantly different in low- and middle-income countries (LMICs) due to resource and healthcare system constraints.

## Objective

The aim of the study was to describe the characteristics of available MoCs for PCOS, their alignment with international guidelines, and their evaluation of outcomes.

## Methods

### Eligibility criteria, information sources, and search strategy

This systematic review was registered on PROSPERO (CRD42022346539). Studies describing MoCs that have more than one specialty in their PCOS management were identified using a search strategy created using MEDLINE limited to English language and human studies. The search strategy was then adapted to different electronic databases. Ovid MEDLINE, All EBM, PsycINFO, Embase, and CINAHL were searched from inception until 11 July 2022. We also included articles identified by experts (CT) that might be relevant to the study. A full search strategy can be found in **Supplementary 1**. Studies were included if they described models of care for PCOS. Any study reported in English with a detailed description of a PCOS MoC was included. Non-evidence-based guidelines, abstracts, study protocols, and clinical trial registrations were excluded. We also excluded MoCs delivered in research settings to minimize care bias. Detailed reasons for exclusion can be found in **Supplementary 2**.

### Study selection

**Figure 3:**
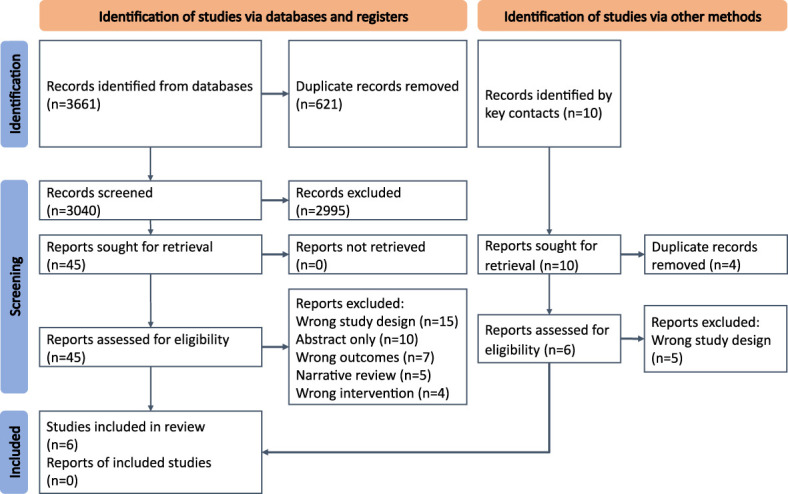
PRISMA chart describing the selection process for our systematic review.

The process for study selection is summarized in **Figure 3**. Titles and abstracts were independently screened by two reviewers (EM and MD) utilizing Covidence software (Covidence systematic review software, Veritas Health Innovation, Melbourne, Australia). Following title and abstract screening, EM and MD screened full texts against the eligibility criteria. Conflicts were resolved following a discussion between the two reviewers and, if needed, by a senior reviewer (PK).

### Data extraction

The researchers (EM, MD, and PK) developed the data extraction template in partnership with the PCOS GDG members (CT, JB, and MT) to ensure relevance. Data extracted included the service name, a detailed description of the MoC and the service, management, and evaluation.

### Assessment of risk of bias

Risk of bias assessments were done using the Monash Centre for Health Research and Implementation (MCHRI) evidence synthesis critical appraisal templates, adapted from the relevant Cochrane critical appraisal tool(s) for mixed-method studies and cross-sectional studies (28). For each study, external and internal validity was assessed to determine the overall risk of bias for that study.

The findings of this review are reported based on the Preferred Reporting Items for Systematic Reviews and Meta-Analyses (PRISMA) guidelines (29). Data are summarized in **Table 1** with a narrative synthesis. Meta-analysis was not performed due to heterogeneity across MoCs. We describe and evaluate each MoC based on the recommendations made by the international evidence-based guideline for assessing and managing PCOS (24).

**Table 1:** Description of included studies.

	Bekx (2010) (30)	Geier (2012) (31)	Boyle (2016) (32)	Torres-Zegarra (2021) (33)	Tay (2021) (34)	Patil (2022) (35)
Characteristics and composition of PCOS MoC						
Country of MoC	United States	United States	Australia	United States	Australia	India
Name of the clinic	Adolescent PCOS clinic at the American Family Children’s Hospital (MoC A)	Adolescent PCOS clinic at the American Family Children’s Hospital (MoC A)	Pilot clinic on Thursday Island, Australia (MoC B)	Multidisciplinary clinic for PCOS at Children’s Hospital Colorado (MoC C)	Monash Health state-wide integrated PCOS service (MoC D)	Integrated multidisciplinary PCOS clinic at Indian Council of Medical Research (ICMR)-National Institute for Research in Reproductive and Child Health (MoC E)
Year of MoC initiation	2005	2005	2012	2012	2017	2016
Members of the multidisciplinary team	Pediatric endocrinologists (×2) Pediatric gynecologist (×1) Reproductive endocrinologist (×1) Nutritionist (×1) Health psychologist (×1)	Pediatric endocrinologists (×2) Pediatric gynecologist (×1) Reproductive endocrinologist (×1) Nutritionist (×1) Health psychologist (×1)	General practitioner (×1) Women’s health nurse (×1) Dietitian (×1) Women’s health worker (×1)	Pediatric Endocrinologist (×1) Gynecologist (×1) Adolescent Medicine Specialist (×1) Dermatologist (×1) (was added to the MoC in 2014) Psychologist (×1) Nutritionist (x1) Exercise Physiologists (x1)	Endocrinologist (×1) Dermatologist (×1) Health coach (×1) Dietician (×1)	Gynecologist (×1) Infertility specialist (×1) Dermatologist (×1) Psychiatrist (×1) Nutritionist (×1) Yoga expert (×1) Counselor (×1)
Services provided in the MoC						
A clear diagnosis of PCOS	Unclear	Participants were given a diagnosis of PCOS based on Rotterdam criteria	Evaluated frequency of Rotterdam criteria met	Requirement for a confirmed diagnosis of PCOS before the first visit; however, unclear according to which criteria	PCOS diagnosis confirmation; however, unclear according to which criteria	PCOS diagnosis confirmation based on the Rotterdam criteria
Cardiometabolic screening, referral, or management	BMI and BMI trends, 2-h OGTT and insulin levels measured	BMI and BMI trends, 2-h OGTT and insulin levels	BMI and BMI trends, blood pressure, 2-h OGTT and insulin levels, HbA1c and lipid profile	BMI, blood pressure, lipid profile and HbA1c	Included screening for long-term health complications but does not describe the components	BMI, waist-hip ratio, blood pressure, ultrasound for non-alcoholic fatty liver disease, lipid profile, 2-h OGTT
Dermatological screening, referral, or management	Hirsutism and acne screening (unspecified screening tool)	Not described	Not described	Hirsutism, acanthosis nigricans and acne screening. Hirsutism with mFG score. Acanthosis and acne were subjective	Medical grade laser for treatment of hirsutism	Acne assessment, Hirsutism with FG score. Dermatologists involved with the management of acne and hirsutism
Education on long-term risk	Not described	Not described	Not described	Group education session on the pathophysiology and medical treatment approaches of PCOS. Educational session by a nutritionist and exercise physiologist on lifestyle recommendations. 30-60 minutes of education to attendees, covering emotional health, bleeding problems, infertility, endometrial protection, and lifestyle factors	Educated attendees regarding the clinical features, diagnosis, complications, and management of PCOS via a group session or printed fact sheets during the first appointment.	Following diagnosis, women were counselled about the condition and the need for an integrated multidisciplinary management
Emotional well-being screening, referral, or management	Unclear	Unclear	Emotional distress screening was with the Kessler Psychological Distress Scale (37)	Psychologists evaluated all patients for mental health symptoms, appetite self-regulation, and emotional eating.	screened using a modified PCOS questionnaire (PCOSQ) (36) Hospital Anxiety and Depression Scale (HADS) (38)	One stop included psychiatrist and psychological counselling that included screening for emotional, and mental health and QoL
Reproductive screening, referral, or management	Unclear	Unclear	lifestyle intervention, metformin prescription, and/or referral to the specialist	screening for endometrial hyperplasia and discussion regarding future infertility issues	Family planning discussion	Has access to gynecologist and infertility specialist
Lifestyle referral or management	Psychologists helped attendees identify barriers that might exist and possible solutions. Nutritionist helped with education on the role of insulin, meal planning, goal setting, and exercise	Psychologists helped attendees identify barriers that might exist and possible solutions. Nutritionist helped with education on the role of insulin, meal planning, goal setting, and exercise	Patients are encouraged to set their own goals including reduction of portion sizes and increasing their walking with follow-up appointments	Exercise physiologists helped describe goals for each exercise and set activities and goals at appointments. Nutritionist helped with monitoring weight trends and education regarding healthy eating	Dietician and/or health coach conducted lifestyle group sessions discussing the importance of a healthy diet and physical activity, personal goal setting, and identification of healthcare barriers	All the women were advised lifestyle modification with diet and exercise in consultation with a nutritionist and yoga expert. Yoga sessions were held as a group activity on the monthly clinic day and women were taught how to practice the specific asanas at home
Evaluations of MoC						
Health professional satisfaction	No	No	Yes	No	No	No
Patient health outcomes	No	Yes	Not described	No	No	Yes
Patient-reported outcomes	No	No	Yes	No	Yes	Yes

## Principal findings

A total of 3,671 articles were identified through title and abstract screening. Of these, 51 articles underwent full-text screening, of which six articles describing five MoCs are included in this report (**Figure 3**). Bekx, Connor, and Allen (30) and Geier, Bekx, and Connor (31) (MoC A) described an adolescent PCOS clinic at the American Family Children’s Hospital, United States; Boyle et al. (32) (MoC B) described a pilot clinic on Thursday Island, Australia; Torres-Zegarra et al. (33) (MoC C) described a multidisciplinary clinic for PCOS at Children’s Hospital Colorado, United States; Tay et al. (34) (MoC D) described the Monash Health state-wide integrated PCOS service, Australia; Patil et al. (35) (MoC E) described an integrated multidisciplinary clinic at the Indian Council of Medical Research, India. Two were mixed-methods studies, and the others were cross-sectional. The objectives of the six articles varied. MoC A, Bekx et al. (30) characterized patients referred to their multidisciplinary clinic, while Geier et al. (31) aimed to examine the impact of MoC A on weight among adolescents with PCOS. Boyle et al. (32) evaluated MoC B based on its fidelity to evidence-based guidelines, barriers, and enablers for women and individuals using their service, and MoC’s ability to meet the needs of women and individuals with PCOS. Torres-Zegarra et al. (33) described the characteristics of patients and patterns of MoC C. Tay et al. (34) evaluated MoC D based on a comprehensive evaluation framework described by Markiewicz and Patrick (39). MoC E described the process of the models of care, including retrospective chart analysis of profiles of women attending the clinic (35). A summary of these MoCs is included in **Table 1**.

### Characteristics and composition of PCOS MoCs

**Figure 4:**
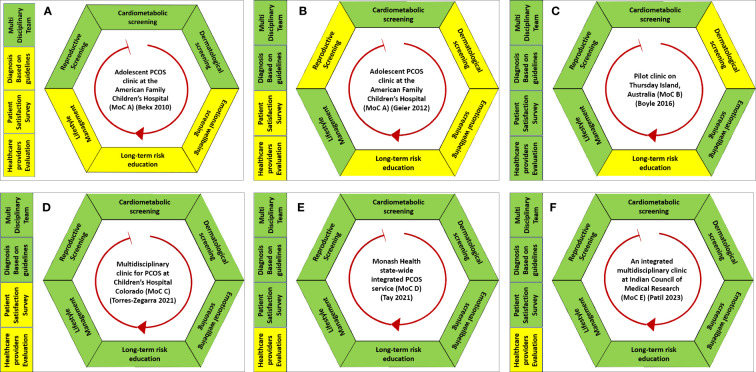
Graphical representation of the five models of care **(A–F)** for polycystic ovary syndrome included in this systematic review. Green represents the services that were provided in a model of care. Yellow represents that the element of MoC was either not reported or unavailable in their MoC.

All the MoCs included had a multidisciplinary approach, but their compositions varied. MoC A was one of the first published MoCs for women and individuals with PCOS (30, 31). Starting in 2005, it had a team of two pediatric endocrinologists, a pediatric gynecologist, a reproductive endocrinologist, a nutritionist, and a psychologist. MoC B, established in 2012, had a general practitioner (GP), a women’s health nurse, a dietician, and a women’s health worker (32). Set up in 2012, MoC C included pediatric endocrinologists, gynecologists/adolescent medicine specialists, psychologists, nutritionists, and exercise physiologists (33). A dermatologist was added to the MoC two years later following patient feedback. MoC D, set up in 2017, was an integrated public multidisciplinary service that comprised specialties including endocrinology, dermatology, health coaching, and dietetics (34). Patients were referred to each specialist clinic when required. MoC E described a one-stop MoC involving a gynecologist, infertility specialist, dermatologist, psychiatrist, nutritionist, yoga expert, and counselor; Women were managed in the clinic regularly (once monthly) (35). Detailed descriptions and characteristics of the MoCs are presented in **Table 1** and **Figure 4**.

### Services provided in the MoC

Three clinics—MoC C, MoC D, and MoC E—reported all aspects of PCOS care in line with the international guidelines. All except MoC A had clear information about the criteria they used for diagnosing PCOS.

### Cardiometabolic disease

All MoCs described some form of cardiometabolic screening, but the content varied. MoC A (30, 31) screened for anthropomorphic characteristics including height, weight, and body mass index (BMI). They also monitored trends in BMI over time to define successful weight loss or gain. A 2-hour oral glucose tolerance test including insulin levels and lipid profile was used to screen for dysglycemia, hyperinsulinemia, and dyslipidemia, respectively. MoC B (32) had all screening done by MoC A with the addition of glycated hemoglobin (HbA1c) and blood pressure measurements. MoC C (33) evaluated BMI, blood pressure, lipid profile, and HbA1c. MoC D (34) included screening for long-term complications. However, the individual components of how this was done were not included in the study. MoC E (35) included BMI screening, waist–hip ratio, ultrasound to assess for non-alcoholic fatty liver disease, and screening for metabolic syndrome, including a 2-hour oral glucose tolerance test, insulin, and lipid profile.

### Lifestyle

All MoCs provided lifestyle interventions, mostly goal setting and education. In MoC A (30, 31), the health psychologist focused on lifestyle changes and helped women and individuals with PCOS identify any barriers that might exist and possible solutions. The nutritionist helped provide education on the role of insulin, meal planning, goal setting, and exercise. In MoC B (32), patients were encouraged to set their own lifestyle goals, which included reducing portion sizes and increasing daily walks. Patients were then asked to attend a follow-up appointment to evaluate their achievements. MoC C (33) included exercise physiologists and nutritionists who provided lifestyle interventions. Exercise physiologists described each exercise and helped set activity goals. A nutritionist helped with monitoring weight trends and provided education regarding healthy eating. Further, health nurses provided 30–60 min of education for women and individuals with PCOS, covering emotional health, bleeding problems, infertility, endometrial protection, and lifestyle. In MoC D (34), a dietician and/or health coach conducted group sessions discussing the importance of a healthy diet and physical activity, personal goal setting, and the identification of healthcare barriers. All women who attended MoC E (35) were advised to modify their lifestyles through diet and exercise with the help of nutritionists and yoga experts.

### Dermatology

All MoCs except MoC B (32) described either screening or treatment for dermatological issues associated with PCOS. MoC A (30) described screening for hirsutism and acne. The screening tools used were not specified in the study. MoC C (33) measured hirsutism using the modified Ferriman–Gallwey (mFG) score. Screening for the presence and severity of acne was done during a physical examination. The presence and absence of acanthosis nigricans, androgenic alopecia, and hidradenitis suppurative were also noted. As for treatment, MoC C (33) used spironolactone, topical treatments, antibiotics, and isotretinoin to manage hirsutism and acne. The dermal clinic integrated into MoC D (34) used a medical-grade laser for hirsutism. MoC E (35) had a dermatologist within the MoC to address acne, oily skin, acanthosis nigricans, and/or hirsutism; however, no specific treatments were described.

### Education on long-term risk

MoCs C and D were the only MoCs that reported education on long-term risks (33, 34). MoC C (33) set up a group education session where women and individuals with PCOS were taught by endocrinologists and gynecologists on the pathophysiology and medical treatment of PCOS. Due to the COVID-19 pandemic, they introduced recorded content for these sessions. MoC D (34) educated women and individuals with PCOS regarding clinical features, diagnosis, complications, and management of PCOS via a group session or printed fact sheets during the first appointment. MoC E (35) counseled women on the condition and the need for integrated multidisciplinary management following the diagnosis of PCOS.

### Emotional well-being and reproductive screening and/or management

Three clinics described the provision of screening for emotional well-being and reproductive health. It was unclear whether MoC A provided emotional and reproductive screening. However, we note that both included health psychologists and a pediatric gynecologist in the clinic. At MoC B (32), emotional distress screening was undertaken with the Kessler Psychological Distress Scale (37), which is a global measure of distress encompassing anxiety and depression items. A psychologist in MoC C (33) evaluated all patients for mental health symptoms, appetite self-regulation, and emotional eating. In MoC D (34), all women and individuals with PCOS were screened using a modified PCOS questionnaire (PCOSQ) (36) and the Hospital Anxiety and Depression Scale (HADS) (38) to evaluate their quality of life and emotional distress, respectively. In MoC B (32), reported infertility treatment included lifestyle intervention, metformin prescription, and/or referral to a specialist. MoC C (33) included screening for endometrial hyperplasia and discussion regarding infertility issues, whereas MoC D (34) included family planning discussion. Women in MoC E (35) were screened for obvious anxiety and/or depression by counselors and addressed by a psychologist or psychiatrist.

## MoC evaluation

MoC evaluation data were organized into three categories: patient outcomes, health professional outcomes, and other outcomes. MoCs B (32) and MoC D (34) were the only studies that reported their MoCs evaluation. MoC B evaluated outcomes from all three categories while MoC D only evaluated patient and other outcomes. Evaluation of patient outcomes was available for MoC A which investigated the impact of their service on BMI. No evaluation outcomes were available for MoC C.

### Patient health outcomes and satisfaction

MoC A (31) evaluated patients’ health outcomes, including improvements in body weight seen in 36% (n = 13/36). Having access to both a psychologist and a dietician was superior for improving weight compared to seeing either alone. MoC B (32) conducted semi-structured interviews and focus groups with women and individuals with PCOS to assess their satisfaction with the clinic. Women and individuals with PCOS found it helpful to have access to this clinic, and they found the staff knowledgeable. Patients also found collaboration with a dietician helpful and valuable in goal setting but suggested more tailored plans and ongoing supervision, indicating insufficiency in what was provided. Overall, 80% (n = 12/15) of patients in MoC D (34) were satisfied with the service. Further semi-structured interviews with women and individuals with PCOS revealed that MoC D covered their multifaceted needs and was effective in providing care and communication. Women and individuals with PCOS also reported a positive impact of this clinic on medical management, symptom severity, their understanding of PCOS, confidence in managing PCOS, and emotional well-being. Suggestions from the interviews included improvements in efficiency, patient communication, resource provision, infrastructure, and awareness of service availability. Patients also suggested more resources to promote self-management. For MoC E (35), telephone feedback was obtained from 155 women who attended the clinic. One year following clinic attendance, 83.8% reported medication adherence, and 52.3% and 46.5% adhered to exercise and dietary interventions, respectively. Sixty-eight percent of women were convinced that a multidisciplinary clinic was helpful in weight reduction and psychological well-being.

### Health professional satisfaction

Health professionals’ satisfaction was investigated by Boyle et al. (2017) in MoC B (32). A survey of service providers found high levels of job satisfaction and professional investment. The service providers saw the absence of a psychologist as a particular problem. Barriers and enablers to clinic sustainability and service delivery were also discussed. Key barriers to sustainability included issues that may arise due to a lack of cover during leave, administrative support, funding, high staff turnover, and system issues. The increased demand for the service, although a strong reason to continue expanding the clinic, was cited as a barrier due to the lack of service providers’ availability.

### Risk of bias in the included studies

Five studies were deemed to have a low risk of bias by the reviewers. One study [MoC A (30)] had a moderate risk of bias due to inadequate information on case selection. Furthermore, the inclusion and exclusion criteria for the study were not described. The detailed risk of bias assessment for each included study is presented in **Supplementary 3**.

## Comparison with existing literature

To date, little progress has been made towards establishing evidence-based PCOS MoCs. Existing MoCs vary considerably in the breadth of multidisciplinary features, with few covering all recommended aspects of care (cardiology, reproduction, dermatology, emotional well-being, lifestyle, and long-term risk). Moreover, it is important to note that some of these studies were not designed to evaluate their MoC, which accounted for the lack of details in each reported MoC. Lack of progress could be because models exist but are not published, health system constraints hinder development (funding, health policy), or there is a lack of know-how about development. Good MoCs for PCOS may exist; however, without their publication, the opportunity to share best practices is lost. We also noted the lack of systematic reporting and evaluation of MoCs in PCOS, and here we have established a structure for capturing and reporting MoC characteristics to support future work. Future research should concentrate on the evaluation of routine MoCs with a focus on patients’ experiences and satisfaction. This would enable the sharing of best practices in the care of women and individuals with PCOS.

The lack of progress in PCOS MoC evaluation in the literature is surprising considering the high prevalence of PCOS as a chronic condition. A systematic review of chronic disease MoCs reported that >90% of their included MoCs (n = 75/77) reported a positive impact on healthcare practices and outcomes (40). A recent systematic review of models of care in primary care for chronic diseases showed improved outcomes for physiological measures of disease, risk behavior, quality of life, health status, patient satisfaction, functional status, and knowledge levels with a model of care (41). Interestingly, the number of studies included was substantially higher in this review in 2014 compared to their previous study in 2006. This shows a growing interest in models of care, especially for complex chronic conditions. However, this is not seen in our review of MoCs in PCOS (in both developed and LMICs). There is a need for and an apparent benefit from multidisciplinary, dedicated one-stop clinics covering all aspects of PCOS, such as the MoCs by Tay et al. (2021) (34) and Torres-Zegarra et al. (2021) (33). This is also in line with the study by Ismayilova and Yaya (2022), where people expressed the need for more PCOS-centric clinics (20). As the management of PCOS is largely individualized due to heterogeneity and a broad range of clinical features, having access to multiple disciplines is important (24). However, our results show that the integration of different disciplines varied considerably, yet four out of the five MoCs showed positive patient and/or healthcare professionals’ satisfaction.

Our systematic review showed that none of the peer-reviewed MoCs is optimized in line with our suggested MoC structure for women and individuals with PCOS. Despite having all the services for women and individuals with PCOS described by Tay et al. (2021) (34), Torres-Zegarra et al. (2021) (33), and Patil et al. (2022) (35), there is currently little evidence of stakeholders’ satisfaction with their MoCs. To ensure the optimization and sustainability of dedicated MoCs, careful design of components of care is important, including a plan for continuous evaluation and monitoring (42). Financial and human resources also play a role in designing such MoCs. Despite the high prevalence and long-term consequences of PCOS, as well as the estimated financial impact of $4.36 billion (43), PCOS receives less than 0.01% of national funding in the US (44). There is a clear need for greater awareness and priority for this condition. This also impacts access to treatment options for PCOS-related symptoms, such as expensive laser hair removal and electrolysis for hirsutism (24, 43, 45). Adequate dermatology management should be provided, as hormonal manipulation with contraceptive pills is not always effective and acne can cause significant mental health issues. Women and individuals with PCOS should be educated about subfertility due to anovulation and, more importantly, referred to a fertility specialist when indicated. As PCOS is also recognized as a metabolic condition, women and individuals with PCOS should be regularly screened for cardiovascular risks and informed of its long-term consequences. Because PCOS is also associated with endometrial cancer, education and public awareness regarding weight loss and progesterone use to reduce endometrial cancer risk are of paramount importance. Emotional well-being screening and appropriate referral are also important for women and individuals with PCOS due to the high prevalence of anxiety, depression, and reduced quality of life that go beyond the physical manifestations of PCOS. All of these would improve self-management strategies for women and individuals with PCOS when coupled with lifestyle interventions that can be provided by healthcare professionals, namely nutritionists, exercise physiologists, and lifestyle coaches. Moreover, it is important to ensure race, culture, and tradition are also factored in when designing an MoC, as these have been shown to influence the differential services received by women and individuals with PCOS (46–50). Other factors that need to be considered include distance to the health facility, affordability that could impact continuity of care, availability of essential treatments, diagnostic tools, trained staff, and coordination of care across public, private, and alternative healthcare providers. These are especially important in LMICs, as shown by the qualitative synthesis from a best-framework synthesis on models of care in LMICs by Lall et al. (51). Moreover, it is also important to consider PCOS MoCs at different life stages, considering the difficulty of diagnosing PCOS in adolescents. These make it vital to involve women, individuals with PCOS, and their families in co-designing services (49).

Many studies have shown that women and individuals with PCOS are generally dissatisfied with their diagnosis experience, the information provided, and the management of their PCOS (18, 19, 21, 22, 52). Patient satisfaction is also an important aspect of healthcare, as it has been shown to affect clinical outcomes and patient retention. Furthermore, patient satisfaction also affects the time and efficacy of healthcare delivery, which is often used as a proxy for the quality of healthcare (53). In addition, healthcare professional satisfaction is key to ensuring the productivity and sustainability of the service (54). In this context, surprisingly few studies focused on PCOS MoC, and most studies did not assess patients’ and healthcare professionals’ satisfaction. We have described satisfaction assessments for two MoCs (32, 34) with positive results. Our findings are like those of two studies describing an MoC based at the Royal Berkshire Hospital, UK, which were not included in this systematic review due to being published as conference abstracts without details of evaluation methodologies (55). An audit was conducted on their MoC to assess the adequacy of investigations and the efficacy of treatment for women and individuals with PCOS attending multidisciplinary clinics. Their patient satisfaction survey showed that 62 out of 63 women found the clinic useful and were happy with the results. They also reported high satisfaction and improved clinical outcomes such as weight loss, menstruation patterns, hirsutism, and physical activity levels (55, 56). A further seven studies that might include PCOS MoC were also excluded from this systematic review because they were abstracts. Hebbar et al. investigated the prevalence of anxiety and body dysmorphia in women and individuals with PCOS attending PCOS specialist clinics in the UK and India (57). The components of their MoC were not described in the abstract (57). Abudu et al. also studied the patient characteristics and subjective improvements in acne for women and individuals attending multidisciplinary PCOS clinics without a description of specialists in the multidisciplinary team (58). Other studies excluded three studies that described either group counseling, self-management, and/or support services for women and individuals with PCOS (59–61).

### Strengths and limitations

The strength of this review includes applying clear definitions of an MoC, which enables the capture of studies aligned with international guidelines. We also established a system to report MoCs; it is important to note that there might be another system that exists for an “optimal MoC.” Our key weakness is related to the limited number of MoCs described internationally, and we note the included MoCs are from two high-income countries—the US and Australia. Therefore, we are unable to generalize our findings to a wider population. Furthermore, due to the design of the included studies, not every component of the MoCs included is captured in our findings. This does not mean that they did not provide the service. Despite only a small number of included studies, this systematic review provides a structured evaluation of the current MoCs for PCOS internationally and further explores their effectiveness.

## Conclusion and implications

There are a limited number of models of multidisciplinary care currently available for PCOS, with a scarcity of data, especially in low- and middle-income countries. Good MoCs may exist, but without their publications, the opportunities to share best practices are lost. Studies on MoC that evaluated patients’ and healthcare professionals’ satisfaction were generally positive. Future work focusing on MoC scale-up should include the development of a best-practice MoC framework, co-designed with women and individuals affected by PCOS across different countries. Alignment with the updated best practice in the 2023 guideline will be important, along with adaptation to the range of health systems and resource settings, and a need for ongoing evaluation and sharing of results to further develop evidence based on real-world experiences.

## Data availability statement

The original contributions presented in the study are included in the article **Supplementary Material**. Further inquiries can be directed to the corresponding author.

## Author contributions

Both EM and MD were involved in all stages of the study and have contributed equally to this work and share the first authorship. KM contributed to the design of the study and data analysis. CT, JB, and MT supervised the data extraction and finalizing of the articles included in the study. Members of the PCOS SEva working group provided substantial contributions to the conception and design of the work and were involved in discussions at all stages of the study. AM and HT were involved in scoping the clinical question and eligibility criteria (PICO), overseeing the review methodology in alignment with approved PCOS guideline evidence synthesis processes, and reviewing and editing the manuscript. PK conceptualized the study and supervised all stages of data collection, analysis, interpretation, and write-up of this study. All authors contributed to the article and approved the submitted version.

## Members of the PCOS SEva working group

Institute of Metabolism and Systems Research, University of Birmingham, Birmingham, United Kingdom: Jameela Sheikh, Meghnaa Hebbar, Halimah Khalil, Kashish Malhotra, Tejal Lathia, Helena K. Gleeson, Lynne Robinson, and Chitra Selvan.

## References

[1] Yildiz BO Bozdag G Yapici Z Esinler I Yarali H. Prevalence, phenotype and cardiometabolic risk of polycystic ovary syndrome under different diagnostic criteria. Hum Reprod (2012) 27(10):3067–73. 10.1093/humrep/des232 22777527

[2] The Rotterdam ESHRE/ASRM‐sponsored PCOS consensus workshop group. Revised 2003 consensus on diagnostic criteria and long‐term health risks related to polycystic ovary syndrome (PCOS). Hum Reprod (2004) 19(1):41–7. doi: 10.1093/humrep/deh098 14688154

[3] Azziz R Carmina E Dewailly D Diamanti-Kandarakis E Escobar-Morreale HF Futterweit W. Positions statement: criteria for defining polycystic ovary syndrome as a predominantly hyperandrogenic syndrome: an androgen excess society guideline. J Clin Endocrinol Metab (2006) 91(11):4237–45. 10.1210/jc.2006-0178 16940456

[4] Barber TM Franks S. Obesity and polycystic ovary syndrome. Clin Endocrinol (2021) 95(4):531–41. 10.1111/cen.14421 33460482

[5] Lim SS Davies MJ Norman RJ Moran LJ. Overweight, obesity and central obesity in women with polycystic ovary syndrome: a systematic review and meta-analysis. Hum Reprod Update (2012) 18(6):618–37. 10.1093/humupd/dms030 22767467

[6] Boureaux MY Talbott EO Kip KE Brooks MM Witchel SF. Risk of T2DM and impaired fasting glucose among PCOS subjects: results of an 8-year follow-up. Curr Diabetes Rep (2006) 6(1):77–83. 10.1007/s11892-006-0056-1 16522285

[7] Rubin KH Glintborg D Nybo M Abrahamsen B Andersen M. Development and risk factors of type 2 diabetes in a nationwide population of women with polycystic ovary syndrome. J Clin Endocrinol Metab (2017) 102(10):3848–57. 10.1210/jc.2017-01354 28938447

[8] Luque-Ramírez M Mendieta-Azcona C Álvarez-Blasco F Escobar-Morreale HF. Androgen excess is associated with the increased carotid intima-media thickness observed in young women with polycystic ovary syndrome. Hum Reprod (2007) 22(12):3197–203. 10.1093/humrep/dem324 17933750

[9] Kumarendran B O’Reilly MW Manolopoulos KN Toulis KA Gokhale KM Sitch AJ. Polycystic ovary syndrome, androgen excess, and the risk of nonalcoholic fatty liver disease in women: a longitudinal study based on a united kingdom primary care database. PloS Med (2018) 15(3):e1002542. 29590099 10.1371/journal.pmed.1002542 PMC5873722

[10] Sarkar M Terrault N Chan W Cedars MI Huddleston HG Duwaerts CC. Polycystic ovary syndrome (PCOS) is associated with NASH severity and advanced fibrosis. Liver Int (2020) 40(2):355. 31627243 10.1111/liv.14279 PMC6980925

[11] Wu J Yao XY Shi RX Liu SF Wang XY. A potential link between polycystic ovary syndrome and non-alcoholic fatty liver disease: an update meta-analysis. Reprod Health (2018) 15(1):77. 29747678 10.1186/s12978-018-0519-2 PMC5946415

[12] Faloia E Canibus P Gatti C Frezza F Santangelo M Garrapa GGM. Body composition, fat distribution and metabolic characteristics in lean and obese women with polycystic ovary syndrome. J Endocrinol Invest (2014) 27(5):424–9. doi: 10.1007/BF03345285 15279073

[13] Brutocao C Zaiem F Alsawas M Morrow AS Murad MH Javed A. Psychiatric disorders in women with polycystic ovary syndrome: a systematic review and meta-analysis. Endocrine (2018) 62(2):318–25. 10.1007/s12020-018-1692-3 30066285

[14] Yin X Ji Y Chan CLW Chan CHY. The mental health of women with polycystic ovary syndrome: a systematic review and meta-analysis. Arch Womens Ment Health (2021) 24(1):11–27. 32514730 10.1007/s00737-020-01043-x

[15] Alur-Gupta S Chemerinski A Liu C Lipson J Allison K Sammel MD. Body-image distress is increased in women with polycystic ovary syndrome and mediates depression and anxiety. Fertil Steril (2019) 112(5):930–938.e1. 31395311 10.1016/j.fertnstert.2019.06.018 PMC6858949

[16] Veltman-verhulst SM Boivin J Eijkemans MJC Fauser BJCM. Emotional distress is a common risk in women with polycystic ovary syndrome: a systematic review and meta-analysis of 28 studies. Hum Reprod Update (2012) 18(6):638–51. 10.1093/humupd/dms029 22824735

[17] Kempegowda P Melson E Manolopoulos KN Arlt W O’Reilly MW. Implicating androgen excess in propagating metabolic disease in polycystic ovary syndrome. Ther Adv Endocrinol Metab (2020) 11:1–24. 2042018820934319. 10.1177/2042018820934319 PMC7315669 32637065

[18] Hillman SC Bryce C Caleyachetty R Dale J. Women’s experiences of diagnosis and management of polycystic ovary syndrome: a mixed-methods study in general practice. Br J Gen Pract (2020) 70(694):e322–9. 10.3399/bjgp20X708881 PMC7065681 32152043

[19] Hoyos LR Ma P Armstrong AA Cheng CY Riestenberg CK Schooler TA. Measures of patient dissatisfaction with health care in polycystic ovary syndrome: retrospective analysis. J Med Internet Res (2020) 22(4):e16541. 32314967 10.2196/16541 PMC7201322

[20] Ismayilova M Yaya S. What can be done to improve polycystic ovary syndrome (PCOS) healthcare? insights from semi-structured interviews with women in Canada. BMC Womens Health (2022) 22(1):157. 35538531 10.1186/s12905-022-01734-w PMC9092874

[21] Gibson-Helm M Tassone EC Teede HJ Dokras A Garad R. The needs of women and healthcare providers regarding polycystic ovary syndrome information, resources, and education: a systematic search and narrative review. Semin Reprod Med (2018) 36(1):35–41. 30189449 10.1055/s-0038-1668086

[22] Ismayilova M Yaya S. “I felt like she didn’t take me seriously”: a multi-methods study examining patient satisfaction and experiences with polycystic ovary syndrome (PCOS) in Canada. BMC Womens Health (2022) 22(1):1–21. doi: 10.1186/s12905-022-01630-3 35197027 PMC8864824

[23] Copp T Muscat DM Hersch J McCaffery KJ Doust J Dokras A. The challenges with managing polycystic ovary syndrome: a qualitative study of women’s and clinicians’ experiences. Patient Educ Couns (2022) 105(3):719–25. 10.1016/j.pec.2021.05.038 34099308

[24] Teede HJ Misso ML Costello MF Dokras A Laven J Moran L. Recommendations from the international evidence-based guideline for the assessment and management of polycystic ovary syndrome. Hum Reprod (2018) 33(9):1602–18. 10.1093/humrep/dey256 PMC6112576 30052961

[25] Davidson P. Beyond the rhetoric: what do we mean by a ‘model of care’? - PubMed. Aust J Adv Nurs (2006) 23(3):47–55. 16568879

[26] Jones AR Tay CT Melder A Vincent AJ Teede H. What are models of care? a systematic search and narrative review to guide development of care models for premature ovarian insufficiency. Semin Reprod Med (2020) 38(4–05):323–30. 10.1055/s-0041-1726131 33684948

[27] Cooney LG Milman LW Hantsoo L Kornfield S Sammel MD Allison KC. Cognitive-behavioral therapy improves weight loss and quality of life in women with polycystic ovary syndrome: a pilot randomized clinical trial. Fertil Steril (2018) 110(1):161–171.e1. 29908771 10.1016/j.fertnstert.2018.03.028 PMC6443091

[28] Moons KGM de Groot JAH Bouwmeester W Vergouwe Y Mallett S Altman DG. Critical appraisal and data extraction for systematic reviews of prediction modelling studies: the CHARMS checklist. PloS Med (2014) 11(10):e1001744. doi: 10.1371/journal.pmed.1001744 25314315 PMC4196729

[29] Page MJ McKenzie JE Bossuyt PM Boutron I Hoffmann TC Mulrow CD. The PRISMA 2020 statement: an updated guideline for reporting systematic reviews. BMJ (2021) 372:n71. 33782057 10.1136/bmj.n71 PMC8005924

[30] Bekx MT Connor EC Allen DB. Characteristics of adolescents presenting to a multidisciplinary clinic for polycystic ovarian syndrome. J Pediatr Adolesc Gynecol (2010) 23(1):7–10. 19648034 10.1016/j.jpag.2009.04.004

[31] Geier LM Bekx MT Connor EL. Factors contributing to initial weight loss among adolescents with polycystic ovary syndrome. J Pediatr Adolesc Gynecol (2012) 25(6):367–70. 10.1016/j.jpag.2012.06.008 23089571

[32] Boyle J Hollands G Beck S Hampel G Wapau H Arnot M. Process evaluation of a pilot evidence-based polycystic ovary syndrome clinic in the Torres strait. Aust J Rural Health (2017) 25(3):175–81. 10.1111/ajr.12288 27086940

[33] Torres-Zegarra C Sundararajan D Benson J Seagle H Witten M Walders-Abramson N. Care for adolescents with polycystic ovary syndrome: development and prescribing patterns of a multidisciplinary clinic. J Pediatr Adolesc Gynecol (2021) 34(5):617–25. 10.1016/j.jpag.2021.02.002 PMC8808364 33794340

[34] Tay CT Pirotta S Teede HJ Moran LJ Robinson T Skouteris H. Polycystic ovary syndrome models of care: a review and qualitative evaluation of a guideline-recommended integrated care. Semin Reprod Med (2021) 39(3–04):133–42. 10.1055/s-0041-1727191 34187051

[35] Patil AD Vaidya RA Begum S Chauhan SL Mukherjee S Kokate PP. An integrated multidisciplinary model of care for addressing comorbidities beyond reproductive health among women with polycystic ovary syndrome in India. Indian J Med Res (2022) 156(3):449–58. 10.4103/ijmr.IJMR_2497_19 PMC10101363 36588359

[36] Williams S Sheffield D Knibb RC. The polycystic ovary syndrome quality of life scale (PCOSQOL): development and preliminary validation. Health Psychol Open (2018) 5(2):1–8. 10.1177/2055102918788195 PMC6053872 30038788

[37] Kessler RC Andrews G Colpe LJ Hiripi E Mroczek DK Normand SLT. Short screening scales to monitor population prevalences and trends in non-specific psychological distress. Psychol Med (2002) 32(6):959–76. 10.1017/s0033291702006074 12214795

[38] Zigmond AS Snaith RP. The hospital anxiety and depression scale. Acta Psychiatr Scand (1983) 67(6):361–70. 10.1111/j.1600-0447.1983.tb09716.x 6880820

[39] Markiewicz A Patrick I. Developing monitoring and evaluation frameworks, by Anne markiewicz and Ian Patrick. Can J Program Eval (2019) 34(1):165–7.

[40] Davy C Bleasel J Liu H Tchan M Ponniah S Brown A. Effectiveness of chronic care models: opportunities for improving healthcare practice and health outcomes: a systematic review. BMC Health Serv Res (2015) 15(1):1–11. doi: 10.1186/s12913-015-0854-8 25958128 PMC4448852

[41] Reynolds R Dennis S Hasan I Slewa J Chen W Tian D. A systematic review of chronic disease management interventions in primary care. BMC Fam Pract (2018) 19(1):11. 29316889 10.1186/s12875-017-0692-3 PMC5759778

[42] Understanding the process to develop a model of care an ACI framework. In: A practical guide on how to develop a model of care at the agency for clinical innovation. (Australia: The Agency for Clinical Innovation). Available at: www.aci.health.nsw.gov.au.

[43] Azziz R Marin C Hoq L Badamgarav E Song P. Health care-related economic burden of the polycystic ovary syndrome during the reproductive life span. J Clin Endocrinol Metab (2005) 90(8):4650–8. 10.1210/jc.2005-0628 15944216

[44] Brakta S Lizneva D Mykhalchenko K Imam A Walker W Diamond MP. Perspectives on polycystic ovary syndrome: is polycystic ovary syndrome research underfunded? J Clin Endocrinol Metab (2017) 102(12):4421–7. 10.1210/jc.2017-01415 29092064

[45] Ndefo UA Eaton A Green MR. Polycystic ovary syndrome: a review of treatment options with a focus on pharmacological approaches. Pharm Ther (2013) 38(6):336. PMC3737989 23946629

[46] Sendur SN Yildiz BO. Influence of ethnicity on different aspects of polycystic ovary syndrome: a systematic review. Reprod BioMed (2021) 42(4):799–818. 10.1016/j.rbmo.2020.12.006 33487557

[47] Paradies Y Ben J Denson N Elias A Priest N Pieterse A. Racism as a determinant of health: a systematic review and meta-analysis. PloS One (2015) 10(9):e0138511. 26398658 10.1371/journal.pone.0138511 PMC4580597

[48] Hendley Y Zhao L Coverson DL Din-Dzietham R Morris A Quyyumi AA. Differences in weight perception among blacks and whites. J Womens Health (2011) 20(12):1805. 10.1089/jwh.2010.2262 PMC3236990 21988528

[49] Lau GM Elghobashy M Thanki M Ibegbulam S Latthe P Gillett CDT. A systematic review of lived experiences of people with polycystic ovary syndrome highlights the need for holistic care and co-creation of educational resources. Front Endocrinol (Lausanne) (2022) 13:1064937/full. doi: 10.3389/fendo.2022.1064937/full 36531482 PMC9755159

[50] PCOS in women of color: its true impact. Available at: https://www.medicalnewstoday.com/articles/made-to-feel-invisible-with-an-invisible-illness-pcos-and-women-of-color.

[51] Lall D Engel N Devadasan N Horstman K Criel B. Models of care for chronic conditions in low/middle-income countries: a ‘best fit’ framework synthesis. BMJ Glob Health (2018) 3(6):e001077. 10.1136/bmjgh-2018-001077 PMC6326308 30687524

[52] Gibson-Helm ME Lucas IM Boyle JA Teedea HJ. Women’s experiences of polycystic ovary syndrome diagnosis. Fam Pract (2014) 31(5):545–9. 10.1093/fampra/cmu028 24925927

[53] Prakash B. Patient satisfaction. J Cutan Aesthet Surg (2010) 3(3):151. 21430827 10.4103/0974-2077.74491 PMC3047732

[54] Dragana N Mirjana A Nikolic M Stancovic A. Job satisfaction in health care workers. Acta Med Med (2008) 47(4):9–12.

[55] Ghosh D Murphy C Elsheikh M. (2005). A 2 year audit of the polycystic ovary syndrome (PCOS) clinic at the royal Berkshire hospital, in: 24th Joint Meeting of the British Endocrine Societies, (UK: Bioscientifica). Available at: https://www.endocrine-abstracts.org/ea/0009/ea0009p79.

[56] Eldridge S Murphy C Elsheikh M. Audit of the polycystic ovary syndrome (PCOS) nurse led weight management clinic. Soc Endocrinol BES (2007) 13.

[57] Hebbar M Shaikh S Zia N Sheikh J Wicks S Jayaprakash S. PCOS SEVa: high prevalence anxiety and body dysmorphia in women with PCOS attending specialist care in the UK and India. Endocrine Abstracts (2022).

[58] Abudu B Golbari N Porter M Reynolds R. Patient characteristics and subjective improvement of acne in a multidisciplinary polycystic ovary syndrome clinic. J Am Acad Dermatol (2019) 81(4):AB99.

[59] Young CC. An integrated self-management intervention for adolescents with polycystic ovary syndrome (2018). Available at: https://www.clinicaltrials.gov/ct2/show/NCT03600337.

[60] Ansari F Hamzehgardeshi Z Elyasi F Moosazadeh M Ahmadi I. The effect of motivational interview based on WhatsApp on the psychological domains of quality of life in infertile women with pcos: a randomized clinical trial. Eur Psychiatry (2021) 64(Suppl 1):S789.

[61] Brooks MA. Online support services: general well-being in women with polycystic ovarian syndrome as a function of the amount of time and satisfaction with online support services. Dissertation Abstracts International: Section B (2005) 66(5-B):281.

