# Supplementary material for: A systematic review of models of care for polycystic ovary syndrome highlights the gap in the literature, especially in developing countries
Source: Front Endocrinol (Lausanne). 2023 Jul 19;14:1217468. doi: 10.3389/fendo.2023.1217468 (PMC10443706; doi:10.3389/fendo.2023.1217468)
Supplement: Supplementary file 1 [file Table_1.docx]

Supplementary Material

A systematic review of models of care for polycystic ovary syndrome highlights a gap in literature, especially in developing countries.

**Eka Melson MSc^1^**^†^**, Meri Davitadze**^†^ **MD^2+^, Aya Mousa PhD^3^, PCOS SEva working group^4^, Helena Teede PhD^3^, Jacky Boivin PhD^5^, Mala Thondan MD^6^, Chau Thien Tay PhD^3^**^††^**, Punith Kempegowda PhD^4, 7^**^††^

^1^University of Leicester, Leicester, United Kingdom

^2^Clinic NeoLab, Tbilisi, Georgia

^3^Monash Centre for Health Research and Implementation, Monash University, Melbourne, Australia

^4^Institute of Applied Health Research, University of Birmingham, Birmingham, United Kingdom

^5^School of Psychology, Cardiff University, Cardiff, Wales, United Kingdom

^6^Harp Family Medical Centre, Melbourne, Australia

^7^Queen Elizabeth Hospital, University Hospitals Birmingham NHS Foundation Trust, Birmingham, United Kingdom

^†^ These authors share first authorship

^††^ These authors share senior authorship

***Correspondence:**Dr Punith Kempegowda (ORCHID: 0000-0003-0954-6512)

Institute of Applied Health Research, University of Birmingham,

Birmingham, United Kingdom

Email: [p.kempegowda@bham.ac.uk](mailto:p.kempegowda@bham.ac.uk)

# Supplementary Data

**Supplementary 1. Search strategy from inception up to 11^th^ July 2022.**

| OVID Medline, All EBM, PsychInfo, EMBASE (results= 2808) | CINAHL (results: 854) |
| --- | --- |
| 1. exp polycystic ovary syndrome/ 2. polycystic ovar*.mp. 3. poly-cystic ovar*.mp. 4. PCO*.mp. 5. (stein-leventhal or leventhal).mp. 6. anovulation/ 7. anovulat*.mp. 8. oligo-ovulat*.mp. 9. oligoovulat*.mp. 10. (ovar* adj5 (sclerocystic or polycystic or poly-cystic or degenerat* or hyperandrogen* or hyper-androgen*)).mp. 11. 1 or 2 or 3 or 4 or 5 or 6 or 7 or 8 or 9 or 10 12. exp Patient Care Team/ 13. exp "Continuity of Patient Care"/ 14. exp Patient Care Planning/ 15. exp Case Management/ 16. exp Patient Care Management/ 17. exp "Delivery of Health Care, Integrated"/ 18. exp Patient-Centered Care/ 19. exp Interdisciplinary Communication/ 20. exp Managed Care Programs/ 21. models, organizational/ 22. (Case-management or care-coordination or care-co-ordination or care-planning).mp. 23. (Multidisciplin* or multi-disciplin* or multiprofessional or multi-professional or interdisciplin* or inter-disciplin* or (multi* and profession*)).mp. 24. ((Interdiscipin* or step* or collaborat* or health or patient* or integrat* or parallel* or stratified or matched or co-managed or comanaged or shared or primary or specialis* or transition* or tailored or bio-psychosocial or contin*or individual* or model*) adj care).mp. 25. ((team-oriented or team oriented) adj (healthcare or health-care or model or practice)).mp. 26. ((inter-professional or interprofessional or integrat* or "home treatment") adj (team or health-care or healthcare or model or practice)).mp. 27. exp Consumer Participation/ 28. exp Self Care/ 29. exp Self Concept/ 30. exp Decision Making, Shared/ 31. exp Decision Support Systems, Clinical/ or decision-support.mp. 32. community informed care.mp. 33. digital first.mp. 34. ((self or self directed or self-directed or self monitor* or self-monitor* or symptom*) adj (care or help or manag* or efficacy or admin* or concept)).mp. 35. or/12-34 36. 11 and 35 37. limit 36 to (english language and humans) | 1. SU polycystic ovary syndrome 2. polycystic ovar* 3. poly-cystic ovar* 4. PCO* 5. Stein-leventhal or Leventhal 6. SU Ovarian cysts 7. SU anovulation 8. Oligo-ovulat* 9. Oligoovulat* 10. ovar* N5 sclerocystic or ovar* N5 polycystic or ovar* N5 poly-cystic or ovar* N5 degenerat* or ovar* N5 hyperandrogen*or ovar* N5 hyperandrogen* 11. S1 OR S2 OR S3 OR S4 OR S5 OR S6 OR S7 OR S8 OR S9 OR S10 12. MH “Consumer Participation +”) 13. (MH "Self Care+") 14. (MH "Self Concept+" 15. ((self or self directed or self-directed or selfmonitor* or selfmonitor* or symptom*) N (care or help or manag* or efficacy or admin* or concept)) 16. Health communication 17. Interdisciplinary communication 18. Patient Care Management 19. Case-management Or care-coordination OR care-co-ordination Or care-planning 20. (MH "Continuity ofPatient Care+") 21. (Multidisciplin* or multi-disciplin* or multiprofessional or multi-professional or interdisciplin* or interdisciplin* or (multi*and profession*)) 22. ((Interdiscipin* or step* or collaborat* or health or patient* or integrat* or parallel* or stratifi ed or matched or comanaged or comanaged or shared or primary or specialis* or transition* or tailored or biopsychosocial orcontin* or individual* ormodel*) N care) 23. (MH "Patient CarePlans+") OR (MH"MultidisciplinaryCare Team+") OR (MH"ComputerizedPatient Record") OR (MH"Patient CenteredCare") 24. (MH "CaseManagement") OR (MH"Decision SupportSystems, Management") 25. (MH "Health Care Delivery+") OR (MH"Health Care Delivery,Integrated") 26. (MH "Managed CarePrograms+") 27. ((team-oriented or team oriented) N (healthcare or healthcare or model or practice)) 28. ((inter-professional or interprofessional or integrat* or "home treatment") N (team or health-care or healthcare or model or practice)) 29. patient centered care or patient-centred care or person centred care or person-centred care 30. shared decision making 31. Decision support or decision-support 32. community informed care 33. digital first 34. S12 OR S13 OR S14 OR S15 OR S16 OR S17 OR S18 OR S19 OR S20 OR S21 OR S22 OR S23 OR S24 OR S25 OR S26 OR S27 OR S28 OR S29 OR S30 OR S31 OR S32 OR S33 35. S11 AND S34 |

**Supplementary 2. Excluded studies following full-text review.**

| **1** | Thomson, R. L., Buckley, J. D., Lim, S. S., Noakes, M., Clifton, P. M., Norman, R. J., & Brinkworth, G. D. (2010). Lifestyle management improves quality of life and depression in overweight and obese women with polycystic ovary syndrome. *Fertility and Sterility*, *94*(5), 1812–1816. https://doi.org/10.1016/J.FERTNSTERT.2009.11.001 | Wrong study design |
| --- | --- | --- |
| **2** | Colwell, K., Lujan, M. E., Lawson, K. L., Pierson, R. A., & Chizen, D. R. (2010). Women’s perceptions of polycystic ovary syndrome following participation in a clinical research study: implications for knowledge, feelings, and daily health practices. *Journal of Obstetrics and Gynaecology Canada : JOGC = Journal d’obstetrique et Gynecologie Du Canada : JOGC*, *32*(5), 453–459. https://doi.org/10.1016/S1701-2163(16)34499-1 | Wrong study design |
| **3** | Cooney, L. G., Milman, L. W., Hantsoo, L., Kornfield, S., Sammel, M. D., Allison, K. C., Epperson, C. N., & Dokras, A. (2018). Cognitive-behavioral therapy improves weight loss and quality of life in women with polycystic ovary syndrome: a pilot randomized clinical trial. *Fertility and Sterility*, *110*(1), 161-171.e1. <https://doi.org/10.1016/J.FERTNSTERT.2018.03.028> | Wrong study design |
| **4** | Jiskoot, G., Dietz de Loos, A., Beerthuizen, A., Timman, R., Busschbach, J., & Laven, J. (2020). Long-term effects of a three-component lifestyle intervention on emotional well-being in women with Polycystic Ovary Syndrome (PCOS): A secondary analysis of a randomized controlled trial. *PloS One*, *15*(6). https://doi.org/10.1371/JOURNAL.PONE.0233876 | Wrong study design |
| **5** | Arentz, S., Smith, C. A., Abbott, J., Fahey, P., Cheema, B. S., & Bensoussan, A. (2017). Combined Lifestyle and Herbal Medicine in Overweight Women with Polycystic Ovary Syndrome (PCOS): A Randomized Controlled Trial. *Phytotherapy Research : PTR*, *31*(9), 1330–1340. https://doi.org/10.1002/PTR.5858 | Wrong study design |
| **6** | Hephzibah Kirubamani, N., & Abraham, M. (2018). Effect of aerobic exercise (Self-help strategy) on the common endocrine problem (PCOS) in late adolescent & young women & impact on their quality of life. *International Journal of Research in Pharmaceutical Sciences*, *9*(4), 1238–1242. https://doi.org/10.26452/IJRPS.V9I4.1663 | Wrong study design |
| **7** | Young, C. C. (2018). *An Integrated Self-Management Intervention for Adolescents With Polycystic Ovary Syndrome*. ClinicalTrials.Gov. https://www.clinicaltrials.gov/ct2/show/NCT03600337 | Abstract only - abstract not found (clinicaltrial.gov) |
| **8** | al Khalifah, R. A., Flórez, I. D., Dennis, B., Neupane, B., Thabane, L., & Bassilious, E. (2015). The effectiveness and safety of treatments used for polycystic ovarian syndrome management in adolescents: a systematic review and network meta-analysis protocol. *Systematic Reviews*, *4*(1). https://doi.org/10.1186/S13643-015-0105-4 | Wrong outcomes |
| **9** | Jiskoot G, Timman R, Beerthuizen A, de Loos AD, Busschbach J, & Laven J. (2019). The impact of a three-component lifestyle intervention on emotional well-being in women with PCOS \| Cochrane Library. *66th Annual Meeting of the Society for Reproductive Investigation*. https://www.cochranelibrary.com/central/doi/10.1002/central/CN-01912734/full | Wrong study design- RCT |
| **10** | Tamilselvi, S., Nalini, S. J., & Vijayaraghavan, R. (2018). Effectiveness of Self Help Strategies {SHS} for PCOS among Young Adult Girls at Selected Colleges at Chennai- Pilot study report. *Research Journal of Pharmacy and Technology*, *11*(7), 3145–3148. https://doi.org/10.5958/0974-360X.2018.00577.2 | Wrong outcomes |
| **11** | Ansari, F., Hamzehgardeshi, Z., Elyasi, F., Moosazadeh, M., & Ahmadi, I. (2021). The effect of motivational interview based on WhatsApp on the psychological domains of quality of life in infertile women with pcos: A randomized clinical trial. *European Psychiatry*, *64*(S1), S789–S789. https://doi.org/10.1192/J.EURPSY.2021.2086 | Abstract only and the data presented does not include description of the model. |
| **12** | Holbrey, S., & Coulson, N. S. (2013). A qualitative investigation of the impact of peer to peer online support for women living with Polycystic Ovary Syndrome. *BMC Women’s Health*, *13*(1), 1–9. <https://doi.org/10.1186/1472-6874-13-51/TABLES/1> | looked only at peer to peer online support which is outside the scope of this review |
| **13** | Moore, A., & Caldwell, J. (n.d.). The importance of collaboration in treating chronic disease: a focus on PCOS and group medical visits. *Women’s Health Care: A Practical Journal for Nurse Practitioners*, *10*(9), 10–18. | abstract only |
| **14** | Tamilselvi, S., & Nalini, S. J. (2020). Effectiveness of self help strategies (SHS) for PCOS on biochemical parameters among young adult girls. *International Journal of Research in Pharmaceutical Sciences*, *11*(3), 3034–3041. <https://doi.org/10.26452/IJRPS.V11I3.2400> | Wrong outcomes |
| **15** | Malik, S. (2016). Indian Fertility Society Good Clinical Practice PCOS Guidelines. *Women’s Health*, *12*(1), 91. https://doi.org/10.2217/WHE.15.96 | Narrative review |
| **16** | van der Spuy, Z. M. (2018). Guidelines for the assessment and management of polycystic ovary syndrome \| Obstetrics and Gynaecology Forum. *Obstetrics and Gynaecology Forum*, *28*(4). https://journals.co.za/doi/10.10520/EJC-1221c30714 | Wrong study design |
| **17** | Lathia, T., Joshi, A., Behl, A., Dhingra, A., Kalra, B., Dua, C., Bajaj, K., Verma, K., Malhotra, N., Galagali, P., Sahay, R., Mittal, S., Bajaj, S., Moorthy, S., Sharma, S., & Kalra, S. (2022). A Practitioner’s Toolkit for Polycystic Ovary Syndrome Counselling. *Indian Journal of Endocrinology and Metabolism*, *26*(1), 17–25. https://doi.org/10.4103/IJEM.IJEM_411_21 | Narrative review |
| **18** | Boyle, J. A., Xu, R., Gilbert, E., Kuczynska-Burggraf, M., Tan, B., Teede, H., Vincent, A., & Gibson-Helm, M. (2018). Ask PCOS: Identifying Need to Inform Evidence-Based App Development for Polycystic Ovary Syndrome. *Seminars in Reproductive Medicine*, *36*(1), 59–65. https://doi.org/10.1055/S-0038-1667187 | Wrong study design – Explores the impact of Ask PCOS app rather than clinic service |
| **19** | Roessler, K. K., Glintborg, D., Ravn, P., Birkebaek, C., & Andersen, M. (2012). Supportive relationships--psychological effects of group counselling in women with polycystic ovary syndrome (PCOS). *Communication & Medicine*, *9*(2), 125–131. https://doi.org/10.1558/CAM.V9I2.125 | examine the psychological impact of a group-oriented approach to disease management and health behaviour in PCOS |
| **20** | Young, C. C., Sagna, A. O., Monge, M., & Rew, L. (2020). A Theoretically Grounded Exploration of Individual and Family Self-Management of Polycystic Ovary Syndrome in Adolescents. *Comprehensive Child and Adolescent Nursing*, *43*(4), 348–362. https://doi.org/10.1080/24694193.2019.1679278 | explored the context and processes of self-management among adolescents, and parents of adolescents, who have PCOS. |
| **21** | Alenzi, E. O. (2021). Cost-effectiveness analysis of polycystic ovary syndrome management and the risk of gestational diabetes in pregnant women: a decision-tree model. *Expert Review of Pharmacoeconomics & Outcomes Research*, *21*(5), 995–999. https://doi.org/10.1080/14737167.2020.1819796 | estimated the cost-effectiveness of metformin to reduce the risk of gestational diabetes mellitus (GDM) in pregnant women with polycystic ovary syndrome (PCOS) |
| **22** | Harrison, E., & Lach, H. W. (2017). Group visits for management of patients with PCOS: A pilot study. *Women’s Healthcare: A Clinical Journal for NPs* , *5*(4), 44–49. https://www.npwomenshealthcare.com/group-visits-patients-pcos/ | aims were (1) to assess the feasibility of implementing group visits in a university setting, (2) to evaluate changes in patient confidence in their ability to self-manage PCOS (self-efficacy), and (3) to ascertain whether participants begin to engage in health-promoting diet and exercise behaviors. |
| **23** | Young, C. C., Rew, L., & Monge, M. (2019). Transition to Self-Management among Adolescents with Polycystic Ovary Syndrome: Parent and Adolescent Perspectives. *Journal of Pediatric Nursing*, *47*, 85. https://doi.org/10.1016/J.PEDN.2019.04.024 | Wrong study design - explored parental and adolescent views of the transition to PCOS self-management |
| **24** | Newland, A. (2019). PCOS management: A multifaceted approach to care. *Nurse Practitioner*, *44*(7), 1–2. https://doi.org/10.1097/01.NPR.0000565124.92469.D2 | Narrative review |
| **25** | Romanski, P., & Stanic, A. K. (2017). Practical Approach to the PCOS Patient. *Current Obstetrics and Gynecology Reports 2017 6:1*, *6*(1), 11–20. https://doi.org/10.1007/S13669-017-0190-6 | Narrative review |
| **26** | Liu, R., Li, M., Wang, P., Yu, M., Wang, Z., & Zhang, G. Z. (2022). Preventive online and offline health management intervention in polycystic ovary syndrome. *World Journal of Clinical Cases*, *10*(10), 3060–3068. https://doi.org/10.12998/WJCC.V10.I10.3060 | Wrong outcomes |
| **27** | Askue B, Buckworth J, Choi-Pearson R, Kiacz ML, Latanick M, & Warbel A. (2007). Encouraging lifestyle modification in the treatment of PCOS in college-age women: a multidisciplinary group approach. . *Women’s Health Care: A Practical Journal for Nurse Practitioners* , *6*(11), 29–29. | Abstract only |
| **28** | Savage, K., Abudu, B., Porter, M., & Reynolds, R. (2019). Factors determining patient-reported improvement in hirsutism in a multidisciplinary PCOS clinic. *Journal of the American Academy of Dermatology*, *81*(4), AB211. <https://doi.org/10.1016/j.jaad.2019.06.776> | Wrong outcome |
| **29** | Tay, C. T., Moran, L. J., Wijeyaratne, C. N., Redman, L. M., Norman, R. J., Teede, H. J., & Joham, A. E. (2018). Integrated Model of Care for Polycystic Ovary Syndrome. *Seminars in Reproductive Medicine*, *36*(1), 86–94. https://doi.org/10.1055/S-0038-1667310 | Narrative review |
| **30** | Auble, B., Elder, D., Gross, A., & Hillman, J. B. (2013). Differences in the management of adolescents with polycystic ovary syndrome across pediatric specialties. *Journal of Pediatric and Adolescent Gynecology*, *26*(4), 234–238. https://doi.org/10.1016/J.JPAG.2013.03.007 | Wrong outcomes |
| **31** | Pirotta, S., Joham, A. E., Moran, L. J., Skouteris, H., & Lim, S. S. (2021). Implementation of the polycystic ovary syndrome guidelines: A mixed method study to inform the design and delivery of a lifestyle management program for women with polycystic ovary syndrome. *Nutrition & Dietetics: The Journal of the Dietitians Association of Australia*, *78*(5), 476–486. https://doi.org/10.1111/1747-0080.12670 | Wrong intervention |
| **32** | Brooks, M. A. (2005). Online support services: General well-being in women with polycystic ovarian syndrome as a function of the amount of time and satisfaction with online support services. *Dissertation Abstracts International: Section B*, *66*(5-B), 2811. | Dissertation abstract. Not enough information to include in the review |
| **33** | Abudu, B., Golbari, N., Porter, M., & Reynolds, R. (2019). Patient characteristics and subjective improvement of acne in a multidisciplinary polycystic ovary syndrome clinic. *Journal of the American Academy of Dermatology*, *81*(4), AB99. https://doi.org/10.1016/j.jaad.2019.06.383 | Abstract only. Wrong outcome |
| **34** | Liao, L. M., Nesic, J., Chadwick, P. M., Brooke-Wavell, K., & Prelevic, G. M. (2008). Exercise and body image distress in overweight and obese women with polycystic ovary syndrome: a pilot investigation. *Gynecological Endocrinology : The Official Journal of the International Society of Gynecological Endocrinology*, *24*(10), 555–561. https://doi.org/10.1080/09513590802288226 | Wrong intervention - looked only at exercise intervention |
| **35** | R, R., JOSE, S. A., K, M., & KM, S. N. (2019). Quality of life in women with polycystic ovarian syndrome: Requisite of clinical pharmacist intervention. *Asian Journal of Pharmaceutical and Clinical Research*, *12*, 100–105. https://doi.org/10.22159/AJPCR.2019.V12I11.34426 | Wrong study design - The study was designed to assess the impact of counseling on QOL in the above patients.not studying effectiveness |
| **36** | Eldridge, S., Murphy, C., & Elsheikh, M. (2007). Audit of the Polycystic Ovary Syndrome (PCOS) Nurse led weight management clinic. *Endocrine Abstracts*. https://www.endocrine-abstracts.org/ea/0013/ea0013p255 | Conference abstract. Insufficient information to include in the review |
| **37** | Ghosh, D., Murphy, C., & Elsheikh, M. (2005). *A 2 year audit of the polycystic ovary syndrome (PCOS) clinic at the Royal Berkshire Hospital \| BES2005 \| 24th Joint Meeting of the British Endocrine Societies \| Endocrine Abstracts*. Endocrine Abstracts. https://www.endocrine-abstracts.org/ea/0009/ea0009p79 | Conference abstract. Insufficient information to include in the review |
| **38** | Gour, A., Dubey, P., Goel, A., & Halder, A. (2022). Remote assessment and reinforcement of patient awareness of role of lifestyle modification and treatment adherence in polycystic ovary syndrome using an online video based educational module. *Journal of the Turkish German Gynecological Association*, *23*(1), 1–7. https://doi.org/10.4274/JTGGA.GALENOS.2021.2021-9-29 | Wrong study outcome - evaluated the role of an online, video-based, structured, educational module in increasing awareness in women with polycystic ovary syndrome (PCOS). |
| **39** | Hebbar, M., Shaikh, S., Zia, N., Sheikh, J., Wicks, S., Jayaprakash, S., Narendran, A., Khalil, H., Gleeson, H., Robinson, L., Ch, J. J., Lathia, T., Selvan, C., Arlt, W., & Kempegowda, P. (2022). PCOS SEVa: High prevalence anxiety and body dysmorphia in women with PCOS attending specialist care in the UK and India. *Endocrine Abstracts*, *81*. https://doi.org/10.1530/ENDOABS.81.EP898 | Conference abstract. Insufficient information, did not study effectiveness |
| **40** | Kazemi, M., McBreairty, L. E., Zello, G. A., Pierson, R. A., Gordon, J. J., Serrao, S. B., Chilibeck, P. D., & Chizen, D. R. (2020). A pulse-based diet and the Therapeutic Lifestyle Changes diet in combination with health counseling and exercise improve health-related quality of life in women with polycystic ovary syndrome: secondary analysis of a randomized controlled trial. *Journal of Psychosomatic Obstetrics and Gynaecology*, *41*(2), 144–153. https://doi.org/10.1080/0167482X.2019.1666820 | Not PCOS MoC |
| **41** | Percy, C. A., Gibbs, T., Potter, L., & Boardman, S. (2009). Nurse-led peer support group: experiences of women with polycystic ovary syndrome. *Journal of Advanced Nursing*, *65*(10), 2046–2055. https://doi.org/10.1111/J.1365-2648.2009.05061.X | explore the experiences of women with polycystic ovary syndrome attending a nurse-led support group. |
| **42** | Schmidt, T. H., Khanijow, K., Cedars, M. I., Huddleston, H., Pasch, L., Wang, E. T., Lee, J., Zane, L. T., & Shinkai, K. (2016). Cutaneous Findings and Systemic Associations in Women With Polycystic Ovary Syndrome. *JAMA Dermatology*, *152*(4), 391–398. https://doi.org/10.1001/JAMADERMATOL.2015.4498 | Wrong study design - To identify cutaneous and systemic features of PCOS that help distinguish women who do and do not meet the diagnostic criteria. |
| **43** | Moradi, F., Ghadiri-Anari, A., Dehghani, A., Vaziri, S. R., & Enjezab, B. (2020). The effectiveness of counseling based on acceptance and commitment therapy on body image and self-esteem in polycystic ovary syndrome: An RCT. *International Journal of Reproductive Biomedicine*, *18*(4), 243. https://doi.org/10.18502/IJRM.V13I4.6887 | Wrong intervention - not multidisciplinary; determined the effectiveness of group counseling based on acceptance and commitment therapy (ACT) on body image and self-esteem in patients with PCOS |
| **44** | Wang, L. H., Liu, Y., Tan, H., & Huang, S. (2022). Transtheoretical model-based mobile health application for PCOS. *Reproductive Health*, *19*(1). https://doi.org/10.1186/S12978-022-01422-W | Wrong intervention - not multidisciplinary; examined the effect of transtheoretical model-based mobile health application intervention program for PCOS. |
| **45** | Young, C. C., Monge, M., Minami, H., Rew, L., Conroy, H., Peretz, C., & Tan, L. (2022). Outcomes of a Mindfulness-Based Healthy Lifestyle Intervention for Adolescents and Young Adults with Polycystic Ovary Syndrome. *Journal of Pediatric and Adolescent Gynecology*, *35*(3), 305–313. https://doi.org/10.1016/J.JPAG.2021.10.016 | Wrong intervention - not multidisciplinary; examined the feasibility, acceptability, and preliminary efficacy of a mindfulness-based healthy lifestyle self-management intervention with adolescents and young adults diagnosed with polycystic ovary syndrome (PCOS). |
| **46** | Atijosan, A. B. (2020). Torturing the helpless: A review of PCOS induced infertility from a gender perspective. *Journal of Gender and Power*, *14*(2), 157–168. https://doi.org/10.2478/JGP-2020-0019 | Wrong study design |
| **47** | Dapherede Otusanya, A. (n.d.). *“If You Never Came in and Saw Me, You Would Probably Be Dead”: Exploring Intercultural Communication and Health Communication Issues Surrounding Pcos*. | Wrong study design- thesis on experiences rather than model of care |
| **48** | Gezer, E., Piro, B., Cantürk, Z., Çetinarslan, B., Sözen, M., Selek, A., Işik, A. P., & Seal, L. J. (2021). The Comparison of Gender Dysphoria, Body Image Satisfaction and Quality of Life Between Treatment-Naive Transgender Males With and Without Polycystic Ovary Syndrome. *Https://Home.Liebertpub.Com/Trgh*. https://doi.org/10.1089/TRGH.2021.0061 | Wrong study design- evaluated the association of oligo-anovulation and/or features of hyperandrogenism with the scores on the Utrecht Gender Dysphoria Scale (UGDS), the Body Image Scale (BIS), and the Short Form-36 Health Survey (SF-36) in treatment-naive trans men with PCOS seeking help for gender transition |
| **49** | Guss, C. E., & Pitts, S. (2018). Remember to Ask About Gender: Management of Polycystic Ovary Syndrome in Transgender Male Adolescents. *Journal of Pediatric and Adolescent Gynecology*, *31*(2), 182–183. https://doi.org/10.1016/j.jpag.2018.02.060 | Wrong study design- looked into experiences of transgender male adolescents in PCOS clinic |

**Supplementary 3. Risk of bias assessment.**

| **Study** | **Design** | **Selection bias** | | **Performance bias** | **Detection bias** | | **Attrition bias** | **Reporting bias** | **Confounding** | | **Other bias** | **ROB score** |
| --- | --- | --- | --- | --- | --- | --- | --- | --- | --- | --- | --- | --- |
|  |  | **Comparable populations** | **Case and controls defined/ representative** | **Groups treated the same** | **Outcome assessors blinded** | **Outcomes measured reliably** | **Dropouts reported** | **Free of selective reporting** | **Groups similar at baseline** | **Adequate statistical analysis** | **Funding/ COI reported** |  |
| Bekx MT, Connor EC, Allen DB. Characteristics of adolescents presenting to a multidisciplinary clinic for polycystic ovarian syndrome. J Pediatr Adolesc Gynecol. 2010 Feb;23(1):7-10. doi: 10.1016/j.jpag.2009.04.004. Epub 2009 Aug 3. PMID: 19648034. | Cross-sectional study | N/A – only one group | No – only included adolescents with PCOS with no diagnostic criteria | N/A – only one group | N/A – only one group | Partial – outcomes including blood results were measured in different labs | No | Yes | N/A – only one group | Yes – descriptive data were used | No | Moderate |
| Geier LM, Bekx MT, Connor EL. Factors contributing to initial weight loss among adolescents with polycystic ovary syndrome. J Pediatr Adolesc Gynecol. 2012 Dec;25(6):367-70. doi: 10.1016/j.jpag.2012.06.008. Epub 2012 Oct 22. PMID: 23089571. | Cross-sectional study | N/A – only one group | Yes – Rotterdam criteria was used for diagnosis of PCOS | N/A – only one group | N/A – only one group | Not reported | Yes | Yes | N/A – only one group | Yes | No | Moderate |
| Torres-Zegarra C, Sundararajan D, Benson J, Seagle H, Witten M, Walders-Abramson N, Simon SL, Huguelet P, Nokoff NJ, Cree-Green M. Care for Adolescents With Polycystic Ovary Syndrome: Development and Prescribing Patterns of a Multidisciplinary Clinic. J Pediatr Adolesc Gynecol. 2021 Oct;34(5):617-625. doi: 10.1016/j.jpag.2021.02.002. Epub 2021 Mar 29. PMID: 33794340; PMCID: PMC8808364. | Cross-sectional study | N/A – only one group | Yes - Patients included any adolescents and young adults attending the clinic. Not definite diagnosis of PCOS needed | N/A – only one group | N/A – only one group | Yes | Yes | Yes | N/A – only one group | Yes | No | Low |
| Patil AD, Vaidya RA, Begum S, Chauhan SL, Mukherjee S, Kokate PP, Joshi BN. An integrated multidisciplinary model of care for addressing comorbidities beyond reproductive health among women with polycystic ovary syndrome in India. Indian J Med Res. 2022 Sep;156(3):449-458. doi: 10.4103/ijmr.IJMR_2497_19. PMID: 36588359. | Cross-sectional study | N/A – only one group | Yes – Rotterdam criteria was used for diagnosis of PCOS | N/A – only one group | N/A – only one group | Yes | No | Yes | N/A – only one group | Yes | No | Low |

| **Study** | **Validity** | | | | **Results** | | | **Value** | **Reporting bias** | **Other bias** | **ROB score** |
| --- | --- | --- | --- | --- | --- | --- | --- | --- | --- | --- | --- |
|  | **Research design appropriate to address the aims** | **Recruitment strategy appropriate to the aims** | **Data collected in a way that addressed the issue** | **Relationship between researcher and participants been adequately considered** | **Ethical issues been**  **taken into consideration** | **Data analysis sufficiently rigorous** | **Clear statement of findings** | **Valuable is the research** | **Free of selective outcome reporting** | **Funding/ COI reported** |  |
| Boyle J, Hollands G, Beck S, Hampel G, Wapau H, Arnot M, Browne L, Teede HJ, Moran LJ. Process evaluation of a pilot evidence-based Polycystic Ovary Syndrome clinic in the Torres Strait. Aust J Rural Health. 2017 Jun;25(3):175-181. doi: 10.1111/ajr.12288. Epub 2016 Apr 18. PMID: 27086940. | Yes | Yes | Yes | Not reported | Yes | Yes | Yes | Yes | Yes | No | Moderate |
| Tay CT, Pirotta S, Teede HJ, Moran LJ, Robinson T, Skouteris H, Joham AE, Lim SS. Polycystic Ovary Syndrome Models of Care: A Review and Qualitative Evaluation of a Guideline-Recommended Integrated Care. Semin Reprod Med. 2021 Jul;39(3-04):133-142. doi: 10.1055/s-0041-1727191. Epub 2021 Jun 29. PMID: 34187051. | Yes | Yes | Yes | Yes | Yes | Yes | Yes | Yes | Yes | No | Low |
